# Quantitative Analysis of Closed Porosity in Direct Resin Composites: A Micro-CT Study on the Influence of Material Type

**DOI:** 10.1155/ijod/3292581

**Published:** 2025-09-22

**Authors:** Enas Mangoush, Lippo Lassila, Pekka K. Vallittu, Sufyan Garoushi

**Affiliations:** ^1^Department of Biomaterials Science and Turku Clinical Biomaterial Center -TCBC, Institute of Dentistry, University of Turku, Turku, Finland; ^2^Wellfare District of South-West Finland, Turku, Finland

**Keywords:** bulk fill, closed porosity, conventional composites, fiber-reinforced composites, flowable composites, micro-cT, packable composites

## Abstract

**Aim:** This study aimed to evaluate and compare the average pores size and closed porosity levels in different types of resin composites using micro-computed tomography (micro-CT).

**Materials and methods:** Ninety specimens (2.5 mm × 4 mm × 4 mm) were prepared using 15 different materials divided into 15 groups (*n* = 6/group). Groups were either conventional packable (Filtek Universal Restorative and G-aenial A'chord), conventional flowable (CLEARFIL MAJESTY ES Flow, G-aenial Flo X, and G-aenial Universal Injectable), bulk fill flowable (SDR flow+ and Filtek Bulk Fill Flowable Restorative), dual-cure (Gradia Core and CLEARFIL DC Core Plus), or fiber-reinforced composites (Nova Pro Flow, Fibrafill DENTIN, everX Flow with either Dentin or Bulk shade, and everX Posterior), in addition to one glass hybrid material (EQUIA Forte HT Fil). All specimens were scanned using a micro-CT machine (Bruker Skyscan 1272), and analyzed for the average size, volume percentage, and distribution of closed porosities. Data was analyzed using Shapiro–Wilk for normality, followed by two-way ANOVA with Tukey's HSD for group comparisons. Pearson correlation assessed the relation between the average size and the percentage of closed pores.

**Results:** The results revealed significant differences in closed porosity levels among the tested groups. Filtek Universal Restorative exhibited the lowest porosity (0.049%), while Fibrafill DENTIN had the highest (4.78%) and the largest average pore size (0.996 mm³). In contrast, A'chord had the smallest average closed pore size (0.017 mm³) (*p*  < 0.05). When the composites were categorized into flowable and packable variants, a significant difference (*p*  < 0.05) was observed in the average percentage of closed pores, with flowable composites showing a lower porosity (0.31%) compared to packable composites (1.25%).

**Conclusion:** The findings highlight the influence of material type on porosity formation, with larger pore sizes correlating with higher closed porosity percentages.

## 1. Introduction

Resin composites have become prevalent in restorative dentistry for several decades due to their esthetic and mechanical properties [1]. However, despite their preferable properties, resin composites still exhibit certain intrinsic limitations, which may relate for instance to the formation of porosities [2]. Porosity in resin composites refers to the spaces, air bubbles, or voids within or at the boundary of the material, expressed as a volume percentage [3]. To the best of our knowledge, direct clinical evidence linking porosity to performance in resin composite restorations is lacking. However, various in vitro studies indicate that pores may raise clinical concerns [4–6]. For instance, porosity levels at the range of 3 vol.% or more can significantly reduce compressive strength and fatigue limits, with pores acting as critical defect points that can lead to fractures [5].

Higher porosity also correlates with increased water absorption, a process facilitated by the diffusion of water molecules into the morphological voids within the material, which can lead to hydrolytic degradation and anisotropic behavior of the restoration [7–9]. This process can separate polymer chains, degrade the polymer-filler interface, and overall weaken the composite structure [8, 9]. Large pores on the restoration surface may increase risks such as secondary caries when located in regions that could affect the bonding properties within the tooth-restoration interface, though the precise impact remains debated [10]. It has also been shown that major air voids cause internal oxygen inhibition of polymerization [11].

Porosities in resin composites may also have implications for biocompatibility and esthetic properties of the restorations [7]. The unreacted monomers in proximity to the voids can potentially be released through solubilization, which may induce inflammatory responses in the surrounding tissues [7, 12].

Studies revealed that porosity in resin composites varies significantly, with these differences attributed to factors such as material handling, the characteristics of the matrix and fillers, and variations in manufacturing conditions, including mixing and injection techniques [13, 14]. Furthermore, the methods used to assess porosity, like destructive sectioning, can influence the accuracy of measurements, particularly in terms of pore size and spatial distribution [3].

Resin composites encompass a diverse range of formulations, each distinguished by specific viscosities and handling characteristics, which range from bulk-fill composites to traditional layering composites [2]. Comparing porosity levels among these categories enhances the clinical relevance of the findings by helping practitioners understand how material type may influence restoration durability and integrity.

The viscosity of each composite is primarily governed by factors such as filler content and the composition of the resin matrix, both of which play critical roles in determining the material's susceptibility to air entrapment and the resulting porosity [13].

This study employs a high-resolution micro-computed tomography (micro-CT) to evaluate the porosity characteristics across a range of resin composites, including conventional, bulk-fill, flowable, packable, short fiber-reinforced, and dual-cure formulations, in addition to a glass hybrid material. By using the nondestructive, three-dimensional imaging capabilities of micro-CT, an acceptable level of precise quantification of porosity concerning average size, proportion relative to the total specimen volume, and spatial distribution within the composite matrix can be achieved. Moreover, it ensures that specimens remain intact and can be reused in other investigations [2, 7, 15].

Through comparative analysis across resin composites of varying structures and consistencies, this investigation aims to clarify the relationship between resin composite structure and porosity, offering insights that may inform the selection and manipulation of resin composites to optimize clinical performance and restoration longevity. The tested null hypotheses were: first, that there is no difference in the closed porosity percentage among the different resin composites; and second, that the acquired closed porosity percentage is not correlated with the consistency of the materials.

## 2. Materials and Methods

### 2.1. Specimen Preparation

**Figure 1:**
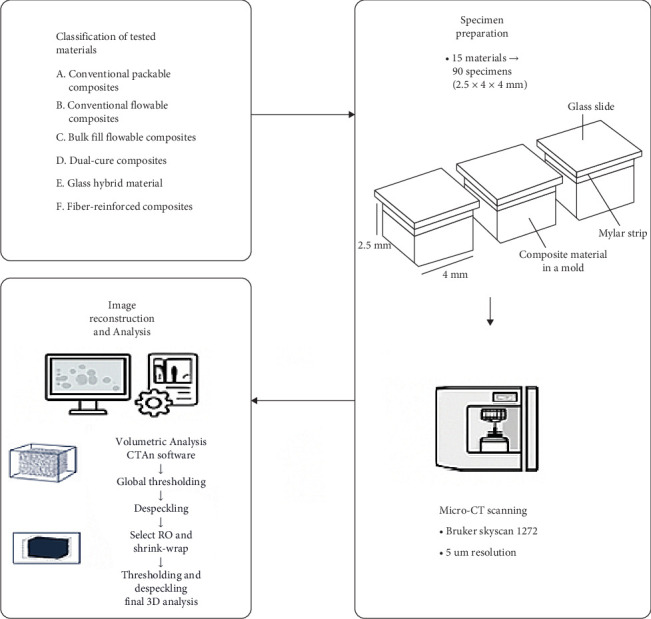
Tested groups, specimens' preparation, scanning, and analysis workflow.

Ninety specimens (2.5 mm × 4 mm × 4 mm) were prepared using 15 materials (Table 1) and divided into 15 groups (*n* = 6/group). The used materials were either conventional packable, flowable, bulk-fill flowable, dual-cure, or fiber-reinforced composites, in addition to one glass hybrid material. Specimens were prepared using putty molds with the specified dimensions. The materials were packed into the molds, and before polymerization, the upper surface was covered with a mylar strip and a glass slide.  Figure 1 shows group classifications of tested materials, specimens' preparation, and analysis workflow.

For bulk-fill resin composites, specimens were prepared in a single 4 mm increment. For layering resin composites, materials were applied in two 2 mm increments, with each increment light-cured for 20 s. A LED light-curing unit (Elipar DeepCure-S, 3M ESPE, Germany) was used to polymerize the resin composites, delivering 1200 mW/cm [2] of intensity, following the manufacturer's instructions and keeping the light tip of the curing device in contact to mylar sheet on top of the resin composite. Before preparing each group, the curing light's intensity was verified using a MARC resin calibrator (BlueLight Analytics, Canada). Specimens were stored dry for 48 h before imaging except for EQUIA Forte HT Fil specimens that were stored after setting in a wet environment for 48 h until imaging. Hydration was maintained to preserve dimensional stability and prevent cracking, which could otherwise compromise the test results.

### 2.2. Micro-CT Imaging and Image Analysis

All specimens were scanned using a high-resolution desktop micro-CT system (Bruker Skyscan 1272, Kontich, Belgium). Each specimen was mounted on a sample rod using transparent orthodontic utility dental wax to stabilize the specimen during the scanning processes. Specimens scanning parameters were as follows: source voltage was 70 kV, source current 142 μA, 5 µm pixel size, rotation at 0.5° step, each specimen was scanned 360° resulting in 720 images. The mean scanning time of each specimen was around 6 min.

Image reconstruction was performed using reconstruction software (NRecon, version 1.7.5.6 Skyscan). To fasten the reconstruction process, a reconstruction engine software (InstaRecon, version: 2.0.4.5 Skyscan) was used. The reconstructed images were analyzed using software (CTAn software, SkyScan) to make volumetric analysis. The processing workflow involved the application of global thresholding, followed by a despeckling procedure to eliminate small objects. Next, the entire specimen was included in the region of interest (ROI), and a shrink-wrap was applied to ensure that only the material of interest was included in the analysis. Subsequently, additional rounds of thresholding and despeckling were performed to further refine the data before conducting the final 3D analysis. After analysis, the resulting data was sent to an Excel spreadsheet for further statistical comparative evaluation. The images were processed for 3D visualization using another software (Skyscan CTVox, Skyscan; The Dataviewer, Skyscan) to assess the 3D spatial distribution of the closed porosity.

### 2.3. Statistical Analysis

The closed porosity data were assessed for normality using the Shapiro–Wilk test. Subsequently, a two-way analysis of variance (ANOVA) was conducted to compare the groups, followed by Tukey's HSD post hoc test for pairwise comparisons. Moreover, groups were divided into two main categories of flowable and packable materials, since the data meets assumptions of normality and homogeneity of variances, an independent sample *t*-test was performed to determine the correlation. The relationship between the average size and the percentage of closed pores regardless of material type was assessed using Pearson correlation analysis, with a significance level set at *p*  < 0.01. All statistical analyses were performed using SPSS software, version 29 (SPSS, IBM Corp., Redmond, WA, USA).

Due to the substantial imaging and computational demands associated with each specimen, a sample size of *n* = 6/group was selected without sample size calculation. This number allowed the detection of statistically significant differences among multiple materials and represented a balance between methodological considerations and statistical reliability.

## 3. Results

**Figure 2:**
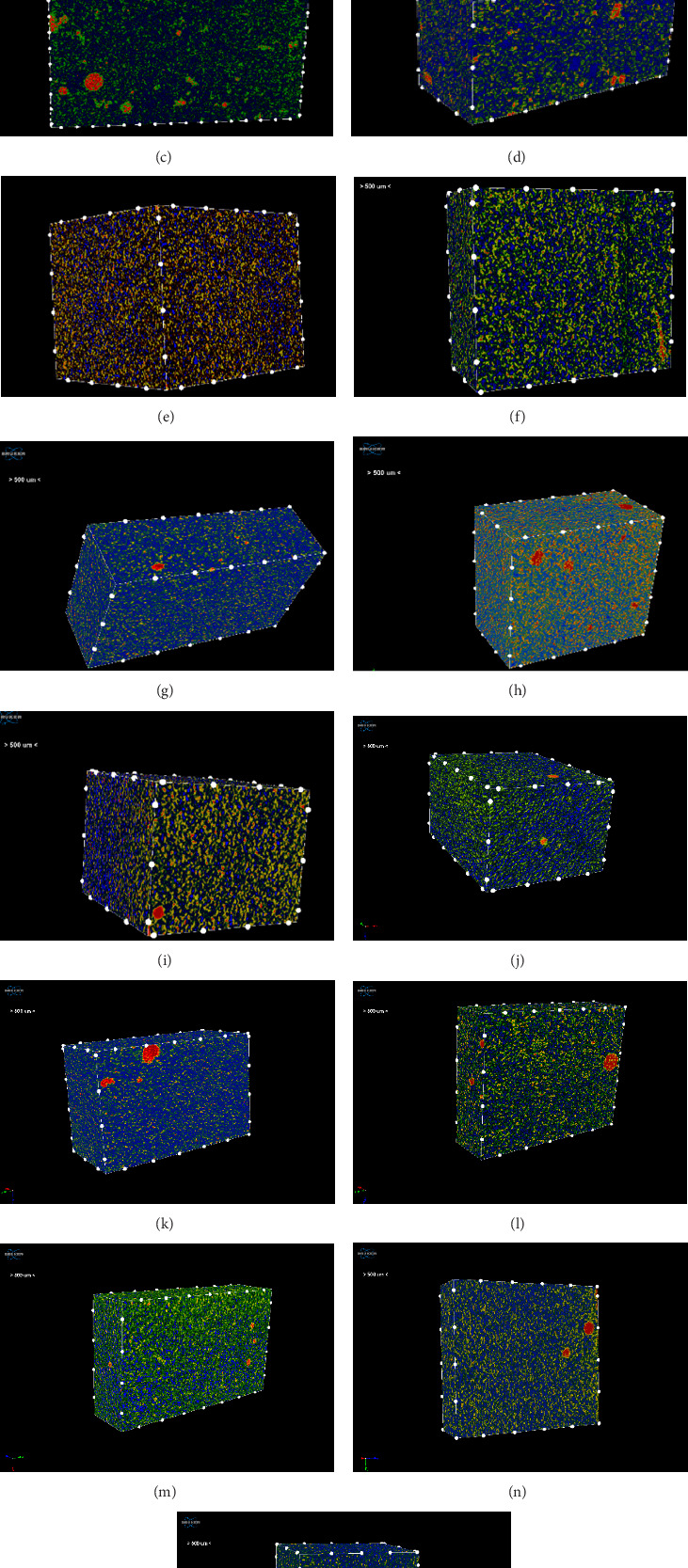
Representative micro-CT 3D images of each tested material. (A) A'chord, (B) Filtek U, (C) Fibrafill, (D) Nova, (E) SDR, (F) Filtek BF, (G) Injectable, (H) CLEARFIL DC, (I) Flo X (J) everX D, (K) everX P, (L) EQUIA, (M) everX B, (N) Gradia, and (O) CLEARFIL F.

**Figure 3:**
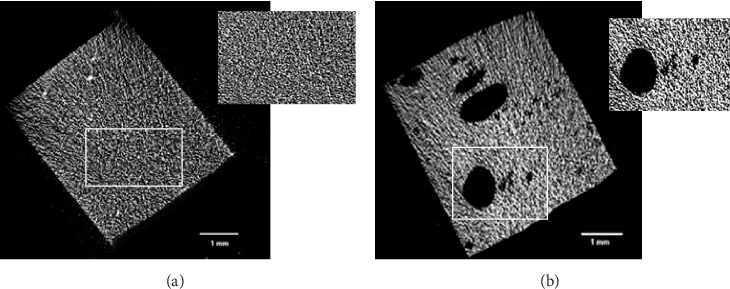
Representative micro-CT 2D images showing the differences between the group that has the lowest (A) Filtek U and the highest (B) Fibrafill percentage of closed pores.

The results of the average size and the volume percentage of the closed pores for each material are shown in Table 2. The conventional packable composites have the lowest average size of closed pores, ranging from 0.017 to 0.018 mm³ for A'chord and Filtek U, respectively, as shown in Figure 2A,B, the pores appear in red color. In contrast, the highest average sizes were observed in two groups of the fiber-reinforced composites, Fibrafill and Nova, at 0.996 and 0.722 mm³, respectively (Figure 2C,D). The pores size of these two materials exhibits substantial variation, ranging from a few micrometers to considerably larger dimensions, likely attributable to air bubbles. For example, pores observed in Fibrafill specimens were found to reach diameters of up to 1 mm^3^ (Figure 3B), similar outliers were also detected in EQUIA, the glass hybrid materials (Figure 2L). The bulk fill flowable composites, SDR and Filtek BF, are also distinguished by having a low average size of closed pores that are homogenously distributed through the whole specimens (Figure 2E,F).

The average pore sizes for the remaining groups, including the other fiber-reinforced composites, dual-cure composites, and conventional flowable composites, were intermediate between the previously mentioned groups (Figure 2G–O, Table 2).

**Figure 4:**
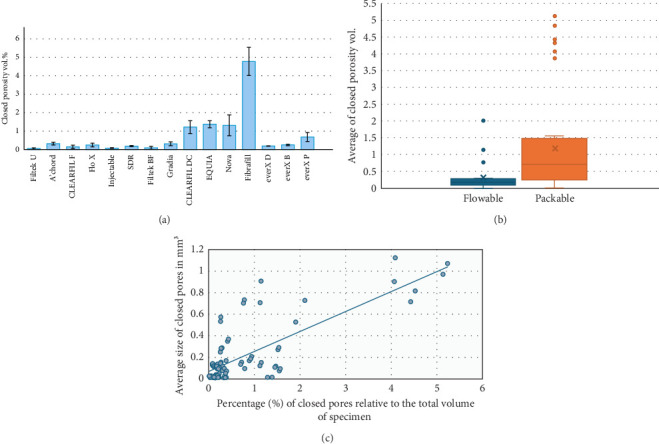
(A) The mean and standard deviation (SD) of the closed porosity (vol.%) for the tested materials. (B) Comparison of the average percentage of closed porosity between flowable and packable resin composites collectively. Error bars represent standard deviations. (C) The correlation between the average size of closed pores and the percentage of closed pores in relation to the total volume of the tested specimen (*R* = 0.778).

The analysis of closed porosity percentage using CTAn software revealed significant variability among the tested groups (Table 2, Figure 4A). As a general finding, the conventional packable, conventional and bulk fill flowable composites have lower porosity percentage compared to the other tested materials; however, some exceptions are present. The mean proportions of closed porosity ranged from 0.049% in the conventional packable composite, Filtek U, to 4.78% in the fiber-reinforced composite, Fibrafill, (Table 2, Figure 2B,C), with the latter showing a statistically significant difference compared to all other groups (*p* <0.05); therefore, the first null hypothesis must be rejected. Representative 2D images (Figure 3A,B) illustrate this difference, with the Fibrafill specimens exhibiting a distinctive sponge-like morphology. The large size and high frequency of the pores in Fibrafill contributed to its significantly higher mean value of closed porosity percentage.

The percentage of closed porosity within the bulk of specimens was notably high in the glass hybrid material (1.375%), which exhibited a statistically significant difference (*p*  < 0.05) compared to all other groups, except for one fiber-reinforced composite, Nova, and one dual-cure composite (CLEARFIL DC), that demonstrated comparable average porosity levels. It is worth mentioning that there is a significant difference (*p*  < 0.05) observed between the two dual-cure composites evaluated in this study, CLEARFIL DC exhibited nearly four times higher closed porosity percentage compared to Gradia specimens. No significant differences were observed between the conventional composites, whether flowable or packable, and the flowable bulk-fill composites (*p*  > 0.05). All these groups were classified by Tukey HSD within the same homogeneous subset.

A direct comparison among the three everX short fiber-reinforced composites groups revealed that the flowable version exhibited a significantly lower average proportion of closed porosities compared to the packable version (*p*  < 0.05) (Figure 4A).

After dividing the materials into two main categories of flowable, and packable, and an independent sample *t*-test was performed, a statistically significant difference in the average percentage of closed pores between flowable and packable resin composites was revealed (t 46.5 = −3.760, *p*  < 0.001) as shown in Figure 4B. The flowable resin composites collectively exhibited a lower mean percentage of closed porosity (mean = 0.309, standard deviation [SD] = 0.432) compared to the packable resin composites (mean = 1.245, SD = 1.563), with a mean difference of −0.9366% (95% confidence interval: −1.4379 to −0.4354%). This finding necessitates the rejection of the second null hypothesis as well.

Figure 4C illustrates a clear positive linear relationship between the average size of closed pores and their overall percentage, as shown by the upward trend in the data points and the fitted trend line. Pearson correlation analysis confirmed this association (*R* = 0.778, *p* < 0.001, *n* = 90), indicating that larger closed pores correspond to a higher proportion of closed porosity in the analyzed materials.

## 4. Discussion

The current study investigated the porosity characteristics of various resin composites, revealing significant differences influenced by material type. Closed pores may act as internal stress concentrators, weakening the material under cyclic loading and potentially increasing the risk of bulk fracture over time. Moreover, excessive porosity may affect water uptake and compromise marginal integrity, increasing susceptibility to microleakage and discoloration, and ultimately reducing the longevity of the restoration [16]. These considerations are important for clinical decision-making, especially when selecting materials for high-stress-bearing restorations. The observed variations in porosity among the tested materials can be attributed to several factors, with material composition being a key determinant.

The filler content, type, size, and organic matrix structure are all factors that may influence the extent of porosity [6, 7, 17]. Attempts to establish a correlation between filler content and the proportion of closed porosities yielded inconclusive results. For example, A'chord exhibits the highest filler content (82 wt.%), followed by everX P and Firafill (78 and 77 wt.%, respectively), yet the percentage of closed porosities within these materials varies significantly (*p*  < 0.05). These findings align with those of Balthazard et al. [7], who also reported inconsistent results when investigating the relationship between filler content and porosity. Consequently, it was concluded that filler content alone cannot adequately explain the observed variations in porosity [7]. Other studies have reported conflicting findings, some have demonstrated a general increase in porosity with a higher filler fraction, indicating a positive correlation between filler weight percentage and porosity [18–20]. Nilsen et al. [3], have shown that flowable composites exhibit reduced porosity levels. This is in accordance with our findings in which flowable materials collectively exhibit a lower mean percentage of closed porosity compared to the packable ones. However, caution should be taken when making generalizations about the consistency, since the observed differences between the flowable and packable variants may be influenced by groups exhibiting extreme results, which could substantially impact the overall findings.

Beyond filler content, several studies have highlighted that variations in particle size and filler morphology can significantly affect the material's microstructure and its ability to trap air during application and curing [15, 21]. In most resin composites, a multimodal particle size distribution is employed, combining coarse and fine particles to enhance the packing density of the inorganic framework, thereby minimizing the likelihood of air entrapment during the preparation and application of the material [22]. Our findings indicate that A'chord group exhibits the smallest average closed pore size among all tested groups, followed by Filtek U and Injectable. Despite variations in filler content by weight, these groups feature the smallest filler sizes within nanometer scale (Table 1), which may contribute to the observed reduction in pore size. These three groups belong to the category of conventional composites applied incrementally, that allows better compaction and may help reduce larger air pockets compared to bulk-fill methods [23, 24]. However, despite its advantages, incremental layering can still introduce micro-bubbles at each step, particularly at composite-tooth or composite–composite interfaces. These voids may act as stress concentrators, compromising the restoration's integrity [25, 26]. Clinicians should therefore employ techniques such as careful syringe placement, slow composite extrusion, and consistent vibration or instrument tapping during each increment to expel trapped air before light curing.

In addition to the previous parameters, the resin matrix structure may influence the porosity levels, and perceiving these parameters interactions is crucial for understanding the overall materials characteristics and behavior. The composition of the resin matrix can affect the material's viscosity, polymerization shrinkage, and overall handling properties, which in turn impact porosity formation [27]. Variations in monomer types and ratios can alter the cross-linking density and the potential for air entrapment during the application process [7, 28]. The reactivity of monomers varies significantly, for instance, the most commonly used monomers reactivity follow this hierarchy: Bis-GMA < Bis-EMA < UDMA < TEGDMA [29]. This variation is attributed to intrinsic molecular characteristics; for example, Bis-GMA exhibits strong intramolecular hydrogen bonding, which leads to high viscosity and consequently reduces both its reactivity and molecular mobility during the polymerization process [29]. While incorporating other monomers with reduced viscosity and without an aromatic core, such as TEGDMA, is known to result in a polymer network exhibiting greater heterogeneity due to their higher flexibility and lower molecular weight. This increased heterogeneity is attributed to the formation of microgel agglomerates within the polymer matrix that can lead to the development of microporosities between polymer clusters [30]. Furthermore, the interaction between the resin matrix and fillers could be one of the factors in determining the porosity values. A less effective coupling, as seen with low-viscosity monomers like TEGDMA, can result in increased porosity at the filler-matrix interfaces [31].

According to our results, Filtek U, Injectable, and Filtek BF have the lowest proportions of closed porosity, they contain monomers of AUDMA, Bis-GMA, Bis-EMA, and UDMA. AUDMA in Filtek U is a type of UDMA monomer (Aromatic UDMA). Due to the rigid aromatic structure, AUDMA typically has a viscosity lies between Bis-GMA and TEGDMA which can lead to better flowability and less porosity [32].

In contrast, Fibrafill contains EBPADMA in addition to the widely used TEGDMA. EBPADMA is a derivative of Bis-GMA modified through the incorporation of ethoxy groups. The addition of these ethoxy groups reduces the viscosity compared to Bis-GMA, which may contribute to higher porosity levels, as it could lead to less effective coupling between the resin matrix and inorganic fillers. Furthermore, the increased flexibility introduced by the ethoxy groups could promote heterogeneity in the polymer network, potentially resulting in greater susceptibility to microporosities at the filler-matrix interface and within the resin matrix itself [33].

When the two dual-cure composites were evaluated, Gradia demonstrated a statistically significant lower volume percentage of closed porosity compared to CLEARFIL DC (Table 2). These materials are typically comprised of a base and a catalyst, dispensed from two separate cartridges. Previous research [7, 34] has suggested that differences in closed porosity percentages of the dual-cure materials may be attributed to variations in the mixing technique or the design of mixing tips. However, both the dual-cure materials tested were processed using an identical auto mixing technique, and comparable mixing tips were employed. This controlled approach eliminates the potential influence of these variables on the observed porosity differences. By excluding mixing-related factors, attention is directed toward other potential determinants of the observed differences, for example, variations in the matrix composition of the two composites could play a role in the results. Differences in polymerization shrinkage, as influenced by distinct resin formulations, are a known contributor to porosity levels [24, 35]. Furthermore, differences in the rate and extent of polymerization between the two materials could exacerbate these effects, leading to divergent porosity profiles [24, 35]. Although both materials exhibit a nearly equivalent filler load, differences in filler type, particle size, morphology, and surface treatment may also contribute to the higher porosity levels observed in CLEARFIL DC. Additionally, variations in the initiator and accelerator systems between the materials may impact curing dynamics, further influencing the final porosity levels.

Upon comparing the fiber-reinforced composites groups, notable differences in porosity proportions were observed (Table 2). The higher porosity percentages in Fibrafill and Nova relative to the everX groups can be attributed to a combination of factors. These factors include the smaller fiber sizes, potential fiber aggregation, challenges in fiber-matrix adhesion, polymerization shrinkage dynamics, and the chemical nature of the fibers. Fibrafill incorporates sub-micron-scale silica fibers (Ø 0.3–1.5 μm, and length 10–50 μm) [36], whereas Nova is reinforced with nanometer-scale hydroxyapatite fibers (Ø 50–200 nm, and length 100–150 μm) [37]. In contrast, everX Flow and everX Posterior utilize micrometer and millimeter-scale E-glass fibers (Table 1). Smaller fibers, such as those in Fibrafill and Nova, exhibit a higher surface area-to-volume ratio, which increases their susceptibility to aggregation during the mixing process. This aggregation can result in the formation of fiber bundles, which act as stress concentrators, leading to increased porosity [36, 38]. Uniform fiber dispersion within the resin matrix is essential for minimizing void formation and mechanical weaknesses. Furthermore, polymerization shrinkage during curing can generate significant stresses within the composite. If the resin matrix fails to achieve adequate adaptation to the fibers or exhibits insufficient wetting, shrinkage may promote the formation of voids and subsequently increase porosity [24, 25]. The chemical nature of hydroxyapatite fibers in Nova Flow may also hinder optimal bonding with the resin matrix compared to glass fibers. Hydroxyapatite fibers are inherently hydrophilic [39], which can compromise their compatibility with hydrophobic resin matrices. This disparity at the fiber-matrix interface can result in suboptimal adhesion and, consequently, elevated porosity levels.

The porosity characteristics can also be influenced by the material's fundamental composition and setting mechanisms. Unlike resin-based composites, which rely on polymerization for structural integrity, glass hybrid materials such as EQUIA undergo an acid–base reaction between fluoroaluminosilicate glass powder and polyacrylic acid in an aqueous environment. This reaction might generate water-filled pores, contributing to higher porosity levels [40, 41].

The positive correlation between larger closed pores and a higher proportion of overall closed porosity suggests that the pore size distribution significantly influences the total porosity within the material. This insight is essential because porosity affects key mechanical and physical properties, such as strength, durability, and resistance to stress. By identifying this relationship, materials structure or processing methods can be better tailored to optimize the balance between porosity and the desired clinical performance.

Notably, discrepancies in reported porosity levels are observed when comparing findings from different studies. For instance, micro-CT-based investigations have reported porosity levels that differ from the results of this study [3, 15, 17, 18]. These variations can be attributed to differences in imaging and analysis parameters, as well as the application and curing techniques employed in each study.

The current study findings suggest that filler content alone does not fully account for the observed variations in closed porosity levels. Moreover, a positive correlation was observed between the average pore size and the percentage of closed porosity, where materials with larger pores exhibited a higher overall proportion of closed porosity.

## 5. Conclusion

Within the limitations of this study, the following conclusion can be drawn:

The overall material structure significantly affects closed porosity levels, with flowable resin composites collectively exhibiting a lower mean percentage of closed porosity. These findings underscore the importance of material selection in optimizing clinical outcomes.

## Figures and Tables

**Table 1:** Materials used in the study.

Material	Abbreviation	Main component	Filler by wt.%	Manufacturer
A. Conventional packable composites				
Filtek Universal Restorative	Filtek U	AUDMA, AFM, diurethane-DMA, and 1,12-dodecane-DMA composed of 20 nm silica, 4–11 nm zirconia, and 100 nm ytterbium trifluoride.	76.5	3 M Dental, St. Paul, MN, USA
G-aenial A'chord	A'chord	Bis-MEPP, glass-filler (300 nm barium glass), and 16 nm (fumed silica) filler size: 16 nm.	82	GC Corp, Tokyo, Japan
B. Conventional flowable composites				
CLEARFIL MAJESTY ES Flow	CLEARFIL F	TEGDMA, hydrophobic aromatic dimethacrylate, silanated barium glass filler, and silanated silica, filler size 0.18–3.5 μm.	75	Kuraray Medical Co
G-aenial Flo X	Flo X	UDMA, dimethacrylate comonomers barium glass fillers in nanometer scale (av. Ø 700 nm).	69	GC Corp, Tokyo, Japan
G-aenial Universal Injectable	Injectable	UDMA, Bis-EMA, methacrylate monomers, silica, and barium glass fillers, filler size 150 nm.	69	GC Corp, Tokyo, Japan
C. Bulk fill flowable composites				
SDR flow+	SDR	Barium-alumino-fluoroborosilicate glass, strontium alumino-fluoro-silcate glass, modified UDMA resin, EBPADMA, TEGDMA, camphorquinone photoinitiator, photoaccelerator, BHT, ultraviolet stabilizer, titanium dioxide, iron oxide pigments, and fluorescing agent.	68	Dentsply DeTrey, Konstanz, Germany
Filtek Bulk Fill Flowable Restorative	Filtek BF	Bis-GMA, UDMA, Bis-EMA and procrylat resins. The fillers are a combination of zirconia/silica with a particle size range of 0.01–3.5 µm and ytterbium trifluoride filler with a range of particle sizes from 0.1 to 5.0 µm.	64.5	3 M Dental, St. Paul, MN, USA
D. Dual-cure composites				
Gradia Core	Gradia	UDMA, NPGDMA, GDMA, TEGDMA, silanated Al–F–silicate glass, amorphous SiO2, TiO2, Fe2O3, MgO, initiators, and accelerators.	75	GC Corp, Tokyo, Japan
CLEARFIL DC Core Plus	CLEARFIL DC	Bis-GMA, TEGDMA, hydrophilic aliphatic dimethacrylate, hydrophobic aromatic dimethacrylate, silanated barium glass filler, silanated SiO2, and Al2O3 filler.	74	Kuraray Medical Co, Tokyo, Japan
E. Glass hybrid material				
EQUIA Forte HT Fil	EQUIA	95% strontium fluoro aluminosilicate glass 5% polyacrylic acid liquid: 40% aqueous polyacrylic acid	NA	GC Corp, Tokyo, Japan
F. Fiber-reinforced composites				
Nova Pro Flow	Nova	Bis-EMA, UDMA, TEGDMA, Barium silicate, amorphous fumed silica, and nanometer scale hydroxyapatite fiber	NA	NANOVA Dental, Columbia, USA
Fibrafill DENTIN	Fibrafill	EBPADMA, UDMA, TEGDMA, short inorganic fibers based on SiO_2_ and Al_2_O_3_ (800 and Ø 0.7 μm), barium glass of different particle size distribution (microhybrid type composite), colloidal silicon dioxide, and submicron silica fibers (<10 wt%)	77	Dentapreg, Brno, Czech Republic
everX Flow Dentin shade	everX D	Bis-EMA, TEGDMA, UDMA, short glass fiber (200–300 μm and Ø7 μm), and barium glass	70	GC Corp, Tokyo, Japan
everX Flow Bulk shade	everX B	Bis-EMA, TEGDMA, UDMA, short glass fiber (200–300 μm and Ø7 μm), and barium glass	70	GC Corp, Tokyo, Japan
everX Posterior	everX P	Bis-EMA, TEGDMA, UDMA, millimeter scale glass fiber, and barium glass	78	GC Corp, Tokyo, Japan

Abbreviations: AFM, addition-fragmentation monomer; AUDMA, aromatic urethane dimethacrylate; Bis-EMA, bisphenol A-ethoxylated dimethacrylate; Bis-GMA, bisphenol A-glycidyl methacrylate; Bis-MEPP, bisphenol A- ethoxylated dimethacrylate phosphate; Diurethane-DMA, diurethane dimethacrylate; EBPADMA, ethoxylated bisphenol A- dimethacrylate; NA, not available; TEGDMA, triethylene glycol dimethacrylate.

**Table 2:** The mean and standard deviation (SD) of the size of closed pores (mm^3^), and total porosity (%) for the tested materials.

Material	Mean (SD) of the average size of closed pores (mm^3^)	Mean^a^ (SD) of closed porosity (vol.%)
Filtek U	0.018 (0.0063)	0.049^**E**^ (0.040)
A'chord	0.017 (0.0094)	0.316^**DE**^ (0.078)
CLEARFIL F	0.266 (0.2079)	0.144^**DE**^ (0.092)
Flo X	0.105 (0.0198)	0.240^**DE**^ (0.098)
Injectable	0.024 (0.0082)	0.076^**E**^ (0.018)
SDR	0.032 (0.011)	0.181^**DE**^ (0.032)
Filtek BF	0.027 (0.012)	0.091^**E**^ (0.082)
Gradia	0.250 (0.01262)	0.308^**DE**^ (0.102)
CLEARFIL DC	0.067 (0.0391)	1.214^**B**^ (0.349)
EQUIA	0.175 (0.0838)	1.375^**B**^ (0.189)
Nova	0.722 (0.0806)	1.310^**B**^ (0.564)
Fibrafill	0.996 (0.0960)	4.780^**A**^ (0.766)
everX D	0.112 (0.0156)	0.187^**DE**^ (0.010)
everX B	0.192 (0.0688)	0.249^**DE**^ (0.039)
everX P	0.136 (0.0604)	0.676^**C**^ (0.247)

^a^Different letters of the mean values of total porosity percentage indicate statistically significant differences among the groups (*p*  < 0.05).

## Data Availability

The data that support the findings of this study are available from the corresponding author upon reasonable request.
